# Validation of the Critical-Care Pain Observation Tool (CPOT) in pediatric patients undergoing orthopedic surgery

**DOI:** 10.1080/24740527.2022.2156332

**Published:** 2023-02-17

**Authors:** Mandy M. J. Li, Don Daniel Ocay, Cynthia L. Larche, Kelsey Vickers, Neil Saran, Jean A. Ouellet, Céline Gélinas, Catherine E. Ferland

**Affiliations:** a Department of Clinical Research, Shriners Hospitals for Children–Canada, Montreal, Quebec, Canada; b Department of Experimental Surgery, McGill University, Montreal, Quebec, Canada; c Department of Pediatric Orthopedics, McGill University, Montreal, Quebec, Canada; d Ingram School of Nursing, McGill University, Montreal, Quebec, Canada; e Alan Edwards Centre for Research on Pain, McGill University, Montreal, Quebec, Canada; f Department of Anesthesia, McGill University, Montreal, Quebec, Canada; g Child Health and Human Development Research Axis, Research Institute–McGill University Health Centre, Montreal, Quebec, Canada

**Keywords:** Critical-Care Pain Observation Tool, pediatric, validation, surgery, inpatient

## Abstract

**Background:**

Postoperative pain cannot be measured accurately among many children with intellectual and developmental disabilities, resulting in underrecognition or delay in recognition of pain. The Critical-Care Pain Observation Tool (CPOT) is a pain assessment tool that has been widely validated in critically ill and postoperative adults.

**Aims:**

The objective of this study was to validate the CPOT for use with pediatric patients able to self-report and undergoing posterior spinal fusion surgery.

**Methods:**

Twenty-four patients (10–18 years old) scheduled to undergo surgery were consented to this repeated-measure, within-subject study. To examine discriminative and criterion validation, CPOT scores and patients’ self-reports of pain intensity were collected prospectively by a bedside rater before, during, and after a nonnociceptive and nociceptive procedure on the day following surgery. Patients’ behavioral reactions were video recorded at the bedside and retrospectively viewed by two independent video raters to examine interrater and intrarater reliability of CPOT scores.

**Results:**

Discriminative validation was supported with higher CPOT scores during the nociceptive procedure than during the nonnociceptive procedure. Criterion validation was supported with a moderate positive correlation between the CPOT scores and the patients’ self-reported pain intensity during the nociceptive procedure. A CPOT cutoff score of ≥2 was associated with the maximum sensitivity (61.3%) and specificity (94.1%). Reliability analyses revealed poor to moderate agreement between bedside and video raters and moderate to excellent consistency within video raters.

**Conclusions:**

These findings suggest that the CPOT may be a valid tool to detect pain in pediatric patients in the acute postoperative inpatient care unit after posterior spinal fusion.

## RÉSUMÉ

**Contexte:** La douleur postopératoire ne peut pas être mesurée avec précision chez de nombreux enfants atteints de déficience intellectuelle et développementale, entraînant ainsi une méconnaissance ou un retard dans la reconnaissance de la douleur. Le *Critical-Care Pain Observation Tool* (CPOT) est un outil d’évaluation de la douleur qui a été largement validé chez les adultes gravement malades et postopératoires.

**Objectifs:** L’objectif de cette étude était de valider le CPOT pour une utilisation auprès de patients pédiatriques capables d’autoévaluation et subissant une chirurgie de fusion vertébrale postérieure.

**Méthodes:** Dans le cadre d’une étude intra-sujet, un consentement à cette mesure répétée a été obtenu pour vingt-quatre patients (10–18 ans) devant subir une intervention chirurgicale. Pour examiner la validation discriminante et de critère, les scores CPOT et les autoévaluations des patients concernant l’intensité de la douleur ont été collectés de manière prospective par un évaluateur au chevet du patient avant, pendant et après une procédure non nociceptive et nociceptive le lendemain de la chirurgie. Les réactions comportementales des patients ont été enregistrées sur vidéo au chevet du patient et visionnées rétrospectivement par deux des évaluateurs vidéo indépendants pour examiner la fiabilité des scores CPOT inter-évaluateurs et intra-évaluateurs.

**Résultats:** La validation discriminante a été confirmée par l’obtention de scores plus élevés à l’échelle CPOT pendant la procédure nociceptive que pendant la procédure non nociceptive. La validation de critère a été confirmée par une corrélation positive modérée entre les scores sur l’échelle CPOT et l’intensité de la douleur autoévaluée par les patients pendant la procédure nociceptive. Un score-seuil ≥ 2 sur l’échelle CPOT a été associé à la sensibilité et la spécificité maximales (61,3 % et 94,1 %, respectivement). Les analyses de fiabilité ont révélé une concordance faible à modérée entre les évaluateurs de chevet et les évaluateurs vidéo, et une cocordance modérée à excellente parmi les évaluateurs vidéo.

**Conclusions:** Ces résultats indiquent que le CPOT peut être un outil valide pour détecter la douleur chez les enfants patients de l’unité de soins hospitaliers postopératoires aigus après une fusion rachidienne postérieure.

## Introduction

The clinical challenge of assessing pain of children and adolescents with neuromuscular diseases, such as patients with cerebral palsy (CP), is their inability to effectively report their pain because of intellectual and developmental disabilities (IDDs). Nevertheless, there is no reason to believe that pain is any less frequent or intense in these patients than in normally developing patients. Patients with CP often require major orthopedic surgeries due to motor impairments secondary to anomalies in the brain leading to muscle imbalance and resulting in severe musculoskeletal complications such as hip dislocation and spinal deformities.^1^ These complications result in great pain that can affect their postoperative care and quality of life. Children and adolescents with CP who undergo major orthopedic surgery experience more complicated and costly hospitalizations than normally developing patients undergoing similar orthopedic surgeries, such as patients with adolescent idiopathic scoliosis.^2^ Patients with CP or adolescent idiopathic scoliosis, a three-dimensional deformity of the spine with pronounced single or double curving of the spine,^3^ may undergo spinal fusion surgery with instrumentation, which is an invasive and extensive surgery such that persistent pain is a common postoperative complication.^4^ Resolution of pain and pain management are of paramount importance in the success of any surgical intervention in these patient populations because pain can lead to negative consequences such as prolonged emotional distress, long-term pain medication usage, and delayed recovery from surgery.^4–7^ A case–control analysis revealed that patients able to self-report pain after spinal fusion surgery received more than twice the amount of opioids compared to a matched group of patients with neuromuscular scoliosis.^8^ This suggests that patients with IDDs may be undertreated postoperatively compared with patients able to self-report their pain.

Even though there has been great improvement over the past years with the validation of pain assessment tools designed for children with IDDs,^9–11^ the multiple levels of cognitive functioning and verbal abilities encountered increase the challenges of providing high-quality pain management, and no single approach for pain assessment fits all children with limited communication skills. Because of the elusive nature of pediatric pain in nonverbal children, therapeutic decisions are frequently based on proxy measures of pain (i.e., based on observations by the parents, nurses, and/or physicians) and revert to a series of trial and error.^12^ The Non-Communicating Children’s Pain Checklist–Postoperative Version,^9^ the revised Face, Legs, Activity, Consolability, Cry Scale,^11,13^ the Pediatric Pain Profile (PPP),^10^ and the Individualized Numeric Rating Scale (INRS)^14^ are available, reliable, and validated pain assessment tools for use in patients with IDDs. However, factors that may limit their clinical utility and their ability to inform effective pain management practices include the complexity of the tool and the interpretation of its score, the time required to perform a pain assessment using such a tool, and the individualization of the tool with input from parents or caregivers.^15^ For example, though the revised Face, Legs, Activity, Consolability, Cry Scale and INRS have a score range that is common in pain measurement (i.e., 0–10), the score range of the Non-Communicating Children’s Pain Checklist–Postoperative Version (0–81) and PPP (0–60) is wide and less intuitive. These broader score ranges may hinder the interpretation of pain intensity.^16^ Moreover, some of the presented tools require considerable preparation time, which may be disadvantageous in an acute postoperative setting. The PPP is several pages long, demands ongoing pain assessments, and requires an interview with the parents to personalize the assessment criteria. Some of the presented tools can be utilized rapidly, such as the INRS with its 1-min observation period and 11-point scale. However, the time required to prepare this fully individualized tool may impede its implementation. Furthermore, there are a few important factors that limit the reliability of individualized tools, notably the possibility of parent or caregiver bias. Relying on parental input assumes that parents or caregivers are able to accurately describe their child’s pain behavior. There are instances where an objective observer is better suited than a parent or caregiver to evaluate a child, such as in cases of Munchausen by proxy or situations in which the parent or caregiver is impaired by mental health or addiction issues.^14^

Taking into account the drawbacks of other observational tools, a promising tool that merits investigation in our population of interest is the Critical-Care Pain Observation Tool (CPOT), which is a behavioral scale initially developed to assess pain in critically ill adults unable to self-report.^17^ This pain assessment tool has been validated in adult postoperative, medical, and trauma intensive care unit patients^18–26^ and healthy adults.^27^ The CPOT is recommended by many experts in pain in this vulnerable population because of its well-established psychometric properties^28,29^ and because it has been validated in different languages.^30^ Moreover, the CPOT has a recommended observation period of 1 min, excludes family input and their potential bias, and has simple scoring, which may require less training.^30^ However, there is still a need to explore its utility in other contexts and populations, such as children and adolescents. To our knowledge, only one study has validated a pediatric version of the CPOT, which made adaptations to existing items and added a fifth one (i.e., consolability), resulting in a score range from 0 to 10.^31^

The objective of this study was to perform an initial validation of the original CPOT^17^ in pediatric patients able to self-report who were undergoing major orthopedic surgery such as spinal fusion surgery prior to its validation in patients with CP undergoing a similar surgery. This study aimed to examine (1) discriminative validation of CPOT scores when exposed to common nonnociceptive and nociceptive procedures, (2) criterion validation of CPOT scores with the patients’ self-reports of pain intensity, and (3) interrater and intrarater reliability of CPOT scores by trained bedside and video raters.

## Materials and Methods

### Design, Setting, and Sample

We conducted a repeated-measure, within-subject study at the Shriners Hospitals for Children–Canada between October 2018 and February 2019, between July 2019 and March 2020, and between October 2020 and January 2021. This study was part of a larger prospective study assessing perioperative pain in children undergoing orthopedic surgery and received ethics approval from the Research Ethics Board of McGill University (A08-M71-14B). Patients were screened in an outpatient spine clinic when the decision to undergo surgery was made. Those eligible were invited to participate in the research study and written informed consent was obtained by a trained research assistant. For patients under the age of 14, written informed assent was obtained and written informed consent was obtained from a parent or legal guardian. The study was conducted in accordance with the Declaration of Helsinki. Inclusion criteria included (1) diagnosed with adolescent idiopathic scoliosis between the ages of 10 and 18 years, (2) scheduled to undergo posterior spinal fusion surgery with instrumentation, (3) able to understand either English or French, and (4) able to self-report. Exclusion criteria included (1) previous major surgery, (2) diagnosis of a major chronic medical condition (American Society of Anesthesiology status III or higher), (3) diagnosis of an intellectual disability that would interfere with the ability to understand questions asked, and (4) diagnosis of a condition that may confound behavior assessment, namely, paralysis or neurological and neuromuscular disorders.

### Procedures

A total of six assessments of the main study variables (CPOT scores and patients’ self-reports of pain intensity) were completed by trained research assistant at the patient’s bedside on the day following surgery. Pain assessments were done before, during, and within 15 min after a nonnociceptive procedure (gentle touch on the forearm) and a nociceptive procedure part of routine nursing care (turning from back to side). These two procedures were selected for the examination of discriminative validation; that is, the ability of the CPOT scores to discriminate between a painful and a nonpainful procedure. Therefore, for each patient, six 1-min assessments were made. First, at each assessment, CPOT scores were obtained by the research assistant based on real-time observations of the patient’s behavioral responses. Then, the research assistant asked patients to self-report pain intensity from 0 (*no pain*) to 10 (*worst pain imaginable*) using the Faces Pain Scale–Revised^32^ for criterion validation. This order was established to minimize the first rater’s bias.

Patients were also video recorded using a video camera set up at the foot of the bed to film the patients’ body movements and a second handheld camera used to film the patients’ face. All videos were retrospectively viewed by a second and third rater (research assistants) to examine interrater reliability of the CPOT scores. Intrarater reliability was also examined by having the same second and third raters view the videos 1 month after completion of their initial rating. All raters underwent a 60-min training session by the tool developer to describe the items and scoring of the CPOT, including practice of rating patient videos created for educational purposes.^33^ Such a procedure for inter- and intrarater reliability testing was successfully used in previous studies with the CPOT.^17^

### Outcome Measures

#### Critical-Care Pain Observation Tool

The CPOT includes four behavioral items: (1) facial expressions, (2) body movements, (3) muscle tension, and (4) vocalization (in nonintubated patients) or compliance with the ventilator (in intubated patients).^17^ The CPOT cannot be used if the patient is unresponsive (e.g., paralyzed, under neuromuscular blocking agents, or heavily sedated). Because the assessments were conducted on the day following surgery when patients were no longer intubated, the behavioral item of vocalization was used. Each behavior was rated on a 0 to 2 scale, resulting in a total score ranging from 0 to 8.^17^ A recent systematic review and meta-analysis revealed that the CPOT has been validated in different adult groups and showed good psychometric properties.^34^ Good (Cronbach’s α >0.70) and acceptable (0.50–0.70) internal consistency was found in most adult studies. Interrater reliability reported with weighted κ and/or intraclass correlation coefficients (ICCs) was observed to be greater than 0.60 in half of the adult studies, with lower values (<0.40) mainly observed at rest. Moreover, the CPOT had a moderate diagnostic accuracy when a cutoff score >2 was used for the presence of pain (area under the curve range 72%–91%, sensitivity range 67%–93%, specificity range 46%–90%).^30,34^ It is important to note that the CPOT is intended to support the detection of the presence of pain by the number and intensity of exhibited behaviors and does not give an indication of pain intensity.

#### Self-Reported Pain Intensity

Participants were asked to rate their pain intensity after each assessment with the Faces Pain Scale–Revised along with a numeric rating scale (NRS; validated in pediatric clinical samples) that ranges from 0 (*no pain*) to 10 (*worst pain imaginable*).^32^

#### Sociodemographic and Medical Variables

Demographic information (age and sex) and clinical data (medical history, surgical variables, and administration of analgesics or sedatives within 4 h prior to data collection) were collected from patients’ electronic medical charts.

### Statistical Analysis

Data were analyzed using R Studio and plotted using Prism v9.^35,36^ Sample size calculation was based on a G*Power 3.1 procedure using exact tests for bivariate normal model correlation.^37^ Based on moderate correlations of *r* = 0.50 obtained in previous studies between CPOT scores and self-reports of pain^17,22^ with an alpha of 0.05 and a power of 80%, a sample size of 29 participants was required. Analyses were based on available data, with no imputation for missing data. Descriptive statistics are presented as means and standard deviations unless otherwise specified. Prior to data analysis, the distributions of total CPOT scores were examined via normal probability plots and by the Shapiro-Wilk test for normality. All total CPOT scores, except for the scores from the bedside rater during the nociceptive procedure, were not normally distributed (test statistic range = 0.47–0.94, *P* < 0.16). Nonparametric tests were used because it was assumed that most of the study data were not normally distributed. To address the first aim, the CPOT scores of all raters were analyzed separately by Friedman tests for the assessments before, during, and after the nonnociceptive and nociceptive procedures followed by post hoc Wilcoxon signed rank tests. The second aim was addressed by performing Mann-Whitney tests and bivariate Spearman correlations for CPOT scores of the bedside rater and patients’ self-reports of pain intensity during the nonnociceptive and nociceptive procedures. In the case of significant tests between the CPOT score and the participant’s self-report, receiver operating characteristic curve analysis was used to evaluate the ability of the scale to classify patients who reported moderate to severe pain (≥4/10) or no to mild pain (0–3/10). An area under the curve of 0.5 suggests that the CPOT is unable to detect moderate to severe pain, 0.70 to 0.80 is considered acceptable, 0.80 to 0.90 is excellent, and 0.90 to 1.00 is outstanding.^38^ The third aim was addressed by calculating two-way random effects model ICCs of the CPOT scores obtained by all three raters at each assessment through the viewing of video recordings for each patient. ICCs <0.5, between 0.5 and 0.75, between 0.75 and 0.9, and >0.9 suggested poor, moderate, good, and excellent reliability, respectively, between and within raters.^39,40^

## Results

Forty-two eligible patients were approached and 12 declined the study because they were uncomfortable with an observer or video recording (*n* = 4), overwhelmed by the study (*n* = 3), or not interested in the study (*n* = 5). Thirty patients consented to participate in the study. However, 1 patient dropped out prior to the assessment due to unanticipated severe pain and 5 patients were excluded due to missing video recordings. Because the clinical utility of the CPOT relies also on its validity and reliability, only the data for 24 patients were analyzed, and their demographic characteristics are presented in Table 1. The ages ranged from 11.4 to 17.8 years, with a mean of 15.6 years. Our sample was split equally regarding the number of males and females. No differences were observed in the CPOT scores during the nonnociceptive procedures between male and female patients (*P* > 0.05). Moreover, no correlation was observed between the CPOT scores and patient age, surgical variables, and analgesic intake 4 h prior to the assessment (*P* > 0.05).

**Table 1.** Study sample demographic characteristics.

Patient characteristics	Total patient sample (*n* = 24)
Demographics	
Age (years), mean (SD)	15.6 (1.9)
Sex (*n*) (female:male)	12:12
Surgical variables, mean (SD)	
Surgery length (min)	243.0 (60.1)
Blood loss (mL)	804.1 (496.7)
Number of fused vertebrae	11.2 (2.5)
Analgesic intake, median (Q1–Q3)	
Equivalents of morphine (mg/kg)	0.09 (0.04–0.19)
Tylenol (mg/kg)	11.41 (0.00–13.97)
Ketorolac (mg/kg)	0.29 (0.00–0.37)

Cumulative analgesic intake was calculated based on the total amount of medication patients received 4 h prior to the start of observations. All opioids were converted to oral morphine equivalents.

### Discriminative Validation of CPOT Scores

**Figure 1.**
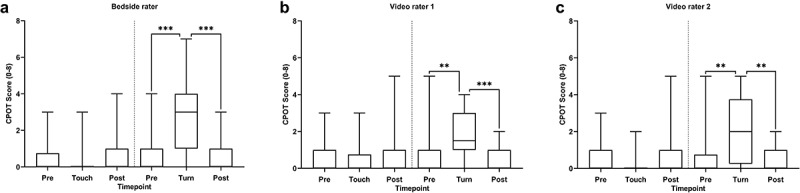
Discriminative validation of the Critical-Care Pain Observation Tool. The CPOT scores for each assessment are summarized for the (a) bedside rater, (b) video rater 1, and (c) video rater 2. The data are presented as the line within the box representing the median, upper and lower limits of the box representing the 25th and 75th percentiles, and the whiskers representing the range. Friedman rank sum tests revealed a significant difference in CPOT scores across the assessments for all raters. Post hoc Wilcoxon tests revealed only a significant difference in CPOT scores before and during the turning procedure and during and postprocedure but not between pre- and post-procedure. *P < 0.05, **P < 0.01, ***P < 0.001.

The frequencies of the individual item scores as well as the median CPOT scores for each assessment and all three raters are presented in Table 2. Friedman test analyses revealed a significant change in CPOT scores before, during, and after touching for the bedside rater (χ^2^ = 6.2, *P* = 0.045) but not for video rater 1 (χ^2^ = 2.6, *P* = 0.273) and video rater 2 (χ^2^ = 2.5, *P* = 0.291). However, post hoc Wilcoxon signed ranked tests with Bonferroni correction (α = 0.017) did not show any significant differences between CPOT scores before, during, and after touching for the bedside rater. Friedman test analyses revealed a significant change in CPOT scores before, during, and after turning for the bedside rater (χ^2^ = 26.3, *P* < 0.001), video rater 1 (χ^2^ = 22.3, *P* < 0.001), and video rater 2 (χ^2^ = 18.2, *P* < 0.001). Post hoc Wilcoxon signed rank tests showed a significant difference only between CPOT scores before and during the turning procedure (*P* < 0.05) and during and postprocedure (*P* < 0.05) but not between pre- and postprocedure (Figure 1).

**Table 2.** CPOT score item frequencies and median total score distributions at each assessment for all raters.

		Pretouch		Touch		Posttouch		Preturn		Turn		Postturn	
	CPOT	*n*	%	*n*	%	*n*	%	*n*	%	*n*	%	*n*	%
Bedside rater													
Facial expression													
Relaxed	0	19	79.2	19	79.2	16	66.7	17	70.8	3	12.5	17	70.8
Tense	1	4	16.7	4	16.7	6	25.0	5	20.8	13	54.2	7	29.2
Grimace	2	1	4.2	1	4.2	2	8.3	2	8.3	8	33.3	0	0.0
Body movement													
Immobile	0	22	91.7	23	95.8	22	91.7	22	91.7	14	58.3	21	87.5
Protection	1	2	8.3	1	4.2	2	8.3	2	8.3	10	41.7	3	12.5
Agitation	2	0	0.0	0	0.0	0	0.0	0	0.0	0	0.0	0	0.0
Vocalization													
No sound/normal	0	23	95.8	23	95.8	23	95.8	23	95.8	13	54.2	24	100.0
Sighing/moaning	1	1	4.2	1	4.2	1	4.2	1	4.2	10	41.7	0	0.0
Crying out/sobbing	2	0	0.0	0	0.0	0	0.0	0	0.0	1	4.2	0	0.0
Muscle tension													
Relaxed	0	21	87.5	22	91.7	18	75.0	18	75.0	8	33.3	20	83.3
Tense	1	3	12.5	2	8.3	6	25.0	6	25.0	13	54.2	4	16.7
Very tense	2	0	0.0	0	0.0	0	0.0	0	0.0	3	12.5	0	0.0
Total CPOT scores													
Median (min–max)		0 (0–3)		0 (0–3)		0 (0–4)		0 (0–4)		3 (0–7)		0 (0–3)	
Self-reported pain													
Median (min–max)		3 (0–8)		3.25 (0–8)		4 (0–8)		4 (0–8)		4.75 (0–9.5)		3 (0–8)	
Moderate–severe		11	45.8	12	50.0	14	58.3	13	54.2	19	79.2	11	45.8
Video rater 1													
Facial expression													
Relaxed	0	19	79.2	21	87.5	18	75.0	19	79.2	10	41.7	19	79.2
Tense	1	5	20.8	3	12.5	5	20.8	4	16.7	7	29.2	5	20.8
Grimace	2	0	0.0	0	0.0	1	4.2	1	4.2	7	29.2	0	0.0
Body movement													
Immobile	0	23	95.8	23	95.8	23	95.8	22	91.7	24	100.0	24	100.0
Protection	1	1	4.2	1	4.2	1	4.2	2	8.3	0	0.0	0	0.0
Agitation	2	0	0.0	0	0.0	0	0.0	0	0.0	0	0.0	0	0.0
Vocalization													
No sound/normal	0	20	83.3	22	91.7	20	83.3	20	83.3	14	58.3	21	87.5
Sighing/moaning	1	4	16.7	2	8.3	4	16.7	4	16.7	9	37.5	3	12.5
Crying out/sobbing	2	0	0.0	0	0.0	0	0.0	0	0.0	1	4.2	0	0.0
Muscle tension													
Relaxed	0	23	95.8	22	91.7	22	91.7	22	91.7	9	37.5	24	100.0
Tense	1	1	4.2	2	8.3	2	8.3	2	8.3	15	62.5	0	0.0
Very tense	2	0	0.0	0	0.0	0	0.0	0	0.0	0	0.0	0	0.0
Total CPOT scores													
Median (min–max)		0 (0–3)		0 (0–3)		0 (0–5)		0 (0–5)		1.5 (0–4)		0 (0–2)	
Video rater 2													
Facial expression													
Relaxed	0	21	87.5	21	87.5	18	75.0	19	79.2	11	45.8	19	79.2
Tense	1	3	12.5	3	12.5	5	20.8	4	16.7	5	20.8	5	20.8
Grimace	2	0	0.0	0	0.0	1	4.2	1	4.2	8	33.3	0	0.0
Body movement													
Immobile	0	22	91.7	23	95.8	23	95.8	22	91.7	23	95.8	24	100.0
Protection	1	2	8.3	1	4.2	1	4.2	2	8.3	1	4.2	0	0.0
Agitation	2	0	0.0	0	0.0	0	0.0	0	0.0	0	0.0	0	0.0
Vocalization													
No sound/normal	0	20	83.3	22	91.7	20	83.3	20	83.3	15	62.5	21	87.5
Sighing/moaning	1	4	16.7	2	8.3	4	16.7	4	16.7	8	33.3	3	12.5
Crying out/sobbing	2	0	0.0	0	0.0	0	0.0	0	0.0	1	4.2	0	0.0
Muscle tension													
Relaxed	0	22	91.7	24	100.0	23	95.8	23	95.8	11	45.8	23	95.8
Tense	1	2	8.3	0	0.0	1	4.2	1	4.2	13	54.2	1	4.2
Very tense	2	0	0.0	0	0.0	0	0.0	0	0.0	0	0.0	0	0.0
Total CPOT scores													
Median (min–max)		0 (0–3)		0 (0–2)		0 (0–5)		0 (0–5)		2 (0–5)		0 (0–2)	

### Criterion Validation of CPOT Scores with Self-Reports of Pain

**Figure 2.**
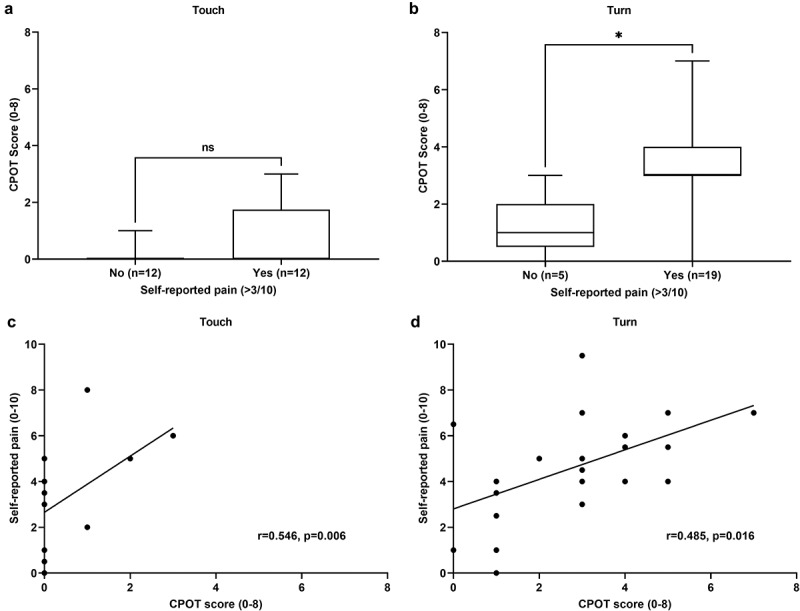
Criterion validation of the Critical-Care Pain Observation Tool. CPOT scores of patients who reported moderate-to-severe pain intensity (≥4/10) during the (a) touching (nonnociceptive) and (b) turning (nociceptive) procedures were compared with patients who reported no to mild pain intensity (0–3/10) with the Mann-Whitney *U* test. The data are presented as the line within the box representing the median, upper and lower limits of the box representing the 25th and 75th percentiles, and the whiskers representing the range. *P < 0.05. Spearman correlation analyses between the CPOT scores and the self-reported pain intensity was conducted during the (c) touching and (d) turning procedures. Data points = individual patients.

Patients’ self-reports of pain were obtained and are described in Table 2. As expected, very few participants reported moderate to severe pain during the touch procedure compared to the turning procedure. Patients who reported moderate to severe pain during the touch procedure did not show a significant difference in their CPOT scores compared to those who reported no to mild pain (*U* = 52.5, *P* = 0.120; Figure 2a). On the other hand, patients who reported moderate to severe pain during the turning procedure showed significantly higher CPOT scores compared to those who reported no to mild pain (*U* = 13.5, *P* = 0.015; Figure 2b). Spearman correlation analyses revealed a moderate positive correlation between self-reports of pain intensity and CPOT scores during the touching (*r* = 0.55, *P* = 0.006) and turning (*r* = 0.49, *P* = 0.016) procedures (Figures 2c and 2d). Receiver operating characteristic curve analysis was then performed to assess the ability of the CPOT to discriminate between patients who reported moderate to severe pain and those who reported no to mild pain during the touching and turning procedures. The area under the curve obtained was 0.78 (95% confidence interval 0.66–0.91, *P* = 0.001), which suggests acceptable discriminate properties. The cutoff point associated with the maximum sensitivity (61.3%) and specificity (94.1%) was observed to be ≥2.

### Reliability of the Raters’ CPOT Scores

#### Interrater Reliability Between Three Trained Raters

ICC was calculated for the CPOT scores collected at each assessment by three trained raters via bedside collection (rater 1) and the viewing of video recordings (raters 2 and 3) for each participant. ICCs ranged from 0.41 to 0.74 (Table 3), indicating poor to moderate agreement between the raters.

**Table 3.** ICC values for all assessments.

	ICC	95% Confidence interval
Interrater reliability (number of raters = 3)		
Touch		
Pre	0.663*	0.453–0.822
During	0.591*	0.364–0.777
Post	0.717*	0.531–0.853
Turn		
Pre	0.740*	0.563–0.866
During	0.569*	0.310–0.770
Post	0.412*	0.167–0.652
Intrarater reliability (video rater 1)		
Touch		
Pre	0.912*	0.807–0.961
During	0.807*	0.603–0.912
Post	0.926*	0.837–0.967
Turn		
Pre	0.929*	0.843–0.969
During	0.929*	0.843–0.969
Post	0.653*	0.347–0.834
Intrarater reliability (video rater 2)		
Touch		
Pre	0.844*	0.673–0.929
During	0.846*	0.677–0.930
Post	0.834*	0.654–0.925
Turn		
Pre	0.855*	0.693–0.934
During	0.701*	0.423–0.859
Post	0.784*	0.562–0.900

*P < 0.001.

#### Intrarater Reliability for Two Trained Video Raters

ICC was also calculated for the CPOT scores collected at each assessment for two trained raters via the viewing of video recordings at least 1 month after the first viewing. This procedure was conducted to avoid raters’ close recall of their initial CPOT scorings. For rater 2, ICCs ranged from 0.65 to 0.93 (Table 3), indicating moderate to excellent consistency across time. For rater 3, ICCs ranged from 0.70 to 0.86 (Table 3), indicating moderate to good consistency across time.

## Discussion

To our knowledge, this study was the first to examine whether the CPOT could successfully capture pain-related behaviors in adolescents with idiopathic scoliosis the day after undergoing posterior spinal fusion surgery. Our findings are consistent with those obtained from previous studies in critically ill adult patients^18–26^ and healthy adults.^27^ During a common nociceptive procedure (i.e., turning) for patients after spinal fusion surgical, patients displayed significant changes in their behavioral CPOT scores compared to a common nonnociceptive procedure (i.e., touching), therefore supporting discriminative validation in this postoperative pediatric patient group. Criterion validation was also supported through the observation of a moderate correlation between the CPOT scores of the bedside rater and the patients’ self-reported pain intensity. Reliability analyses revealed poor to moderate agreement between the bedside and video raters and moderate to excellent consistency within the video raters. Based on these findings, the CPOT may be valid for use in this specific postoperative pediatric group.

In the current study, despite patients displaying an overall increase in their behavioral CPOT scores during the turning procedure, which is known to cause pain after spinal fusion surgery, wide ranges of CPOT scores and self-reported pain intensity were observed. Seven patients demonstrated behavioral CPOT scores <2, and six patients self-reported no to mild pain intensity (i.e., <4 out of 10). It is important to note that the patients still had access to their patient-controlled analgesia morphine pump the day following their surgery, which may have caused some of them to display fewer behavioral changes and self-report mild pain. Nevertheless, the surgical incision was on the back of the patients, which caused the majority of them (79.2%) to self-report moderate to severe pain intensity during turning. It is possible that another common recovery mobilization procedure such as standing would have been more painful in these patients due to the activation of spinal muscles, unlike turning, which only involves moving patients from one side to another while in bed.

Our findings indicate that a CPOT cutoff of ≥2 showed acceptable sensitivity and excellent specificity, which is similar to cutoff thresholds observed in adult postoperative patients in the intensive care unit.^17–26^ Our results suggest that the CPOT can potentially be used in postoperative adolescent patients. The high specificity could help clinicians adequately identify patients in pain and avoid administering analgesics to patients without pain who do not need any. However, half of the patients reported moderate to severe pain during the touch procedure but did not exhibit pain behaviors (median CPOT score = 0). Therefore, although the CPOT may be used to detect pain during painful procedures, it may be difficult to identify pain in patients at rest or during routine care procedures that may not be painful. Further testing and validation are needed to determine whether the CPOT can be used in nonverbal postoperative pediatric patients.

Our results demonstrated moderate interrater reliability during all assessments except for postturning, which demonstrated poor reliability despite a standardized training session for all raters. Poor interrater reliability may have been because the video recordings captured only a window of time lacking contextual data, unlike real-time observations.^41^ Previous studies in adult populations have shown good to excellent interrater reliability before, during, and after nonnociceptive and nociceptive procedures.^18,22^ Knowing that cognitive biases may change in pain observation over time and that there was gap in patient recruitment and testing due to the global pandemic, revision training sessions may have been beneficial to decrease interrater variability. However, moderate to excellent intrarater reliability was observed for two independent video raters during all assessments when video recordings were viewed 1 month after the initial viewing. In the current study, with minimal standardized training,^33^ the CPOT was shown to have moderate to excellent reliability within and between raters and thus could be consistently used.

Our study examined discriminative and criterion validation and inter- and intrarater reliability of the CPOT for the detection of acute postoperative pain in adolescents with idiopathic scoliosis who underwent posterior spinal fusion surgery. Although acceptable results of sensitivity and specificity were obtained, the generalizability of our findings to all pediatric orthopedic surgery patients should be interpreted considering certain limitations. This study had a relatively small sample size with a specific subpopulation of pediatric patients undergoing orthopedic surgery. Moreover, due to unforeseen circumstances, the final sample size was less than required to achieve a power of 80%. Another limitation of the study is the study design, including a nonnociceptive procedure and a nociceptive procedure that consisted of the passive turning of the patient. It is unknown whether the CPOT tool can detect evoked pain through active movement or whether it is sensitive to analgesia administration. Further research with larger sample sizes and in various pediatric contexts, such as evoked nociceptive procedures, after postoperative analgesia administration, and nonverbal children and adolescents, is still needed.
